# Prolonged Outbreak of Carbapenem and Colistin-Resistant *Klebsiella pneumoniae* at a Large Tertiary Hospital in Brazil

**DOI:** 10.3389/fmicb.2022.831770

**Published:** 2022-03-09

**Authors:** Verônica França Diniz Rocha, Matheus Sales Barbosa, Helena Ferreira Leal, Giulyana Evelyn Oliveira Silva, Nabila Monalisa Mendes Dantas Sales, Adriano de Souza Santos Monteiro, Jailton Azevedo, Allan Roberto Xavier Malheiros, Ledilce Almeida Ataide, Beatriz Meurer Moreira, Mitermayer Galvão Reis, Fabianna Márcia Maranhão Bahia, Joice Neves Reis

**Affiliations:** ^1^Laboratory of Pathology and Molecular Biology (LPBM), Gonçalo Moniz Research Institute, Oswaldo Cruz Foundation, Candeal, Brazil; ^2^Faculdade de Farmácia, Universidade Federal da Bahia, Salvador, Brazil; ^3^Secretaria da Saúde do Estado da Bahia, Salvador, Brazil; ^4^Instituto de Microbiologia Paulo de Góes, Universidade Federal Do Rio de Janeiro, Rio de Janeiro, Brazil; ^5^Faculdade de Medicina da Bahia, Universidade Federal da Bahia, Salvador, Brazil; ^6^Yale School of Public Health, Yale University, New Haven, CT, United States

**Keywords:** colistin-resistant *Klebsiella pneumoniae*, carbapenem-resistant *Klebsiella pneumoniae*, multidrug-resistant gram-negative bacteria, outbreak, *Enterobacterales* infections

## Abstract

Multidrug-resistant gram-negative bacteria, such as carbapenem and colistin-resistant *Klebsiella pneumoniae* (ColR-CRKP), represent a major problem for health systems worldwide and have high lethality. This study investigated the genetic relationship, antimicrobial susceptibility profile, and resistance mechanisms to ColR-CRKP isolates from patients infected/colonized in a tertiary hospital in Salvador, Bahia/Brazil. From September 2016 to January 2018, 46 patients (56 ColR-CRKP positive cultures) were enrolled in the investigation but clinical and demographic data were obtained from 31 patients. Most of them were men (67.7%) and elderly (median age of 62 years old), and the median Charlson score was 3. The main comorbidities were systemic arterial hypertension (38.7%), diabetes (32.2%), and cerebrovascular disease (25.8%). The average hospitalization stay until ColR-CRKP identification in days were 35.12. A total of 90.6% used mechanical ventilation and 93.7% used a central venous catheter. Of the 31 patients who had the data evaluated, 12 had ColR-CRKP infection, and seven died (58.4%). Previous use of polymyxins was identified in 32.2% of the cases, and carbapenems were identified in 70.9%. The minimum inhibitory concentration (MIC) for colistin was > 16 μg/mL, with more than half of the isolates (55%) having a MIC of 256 μg/mL. The *bla*_*KPC*_ gene was detected in 94.7% of the isolates, *bla*_*NDM*_ in 16.0%, and *bla*_*GES*_ in 1.7%. The *bla*_*OXA–*48_, *bla*_*VIM*,_ and *bla*_*IMP*_ genes were not detected. The *mcr-1* test was negative in all 56 isolates. Alteration of the *mgrB* gene was detected in 87.5% (*n* = 49/56) of the isolates, and of these, 49.0% (24/49) had alteration in size probably due to IS*903B*, 22.4% (11/49) did not have the *mgrB* gene detected, 20.4% (10/49) presented the IS*903B*, 6.1% (3/49) had a premature stop codon (Q30*), and 2.1% (1/49) presented a thymine deletion at position 104 – 104delT (F35fs). The PFGE profile showed a monoclonal profile in 84.7% of the isolates in different hospital sectors, with ST11 (CC-258) being the most frequent sequence type. This study presents a prolonged outbreak of ColR-CRKP in which 83.9% of the isolates belonged to the same cluster, and 67.6% of the patients evaluated had not used polymyxin, suggesting the possibility of cross-transmission of ColR-CRKP isolates.

## Introduction

Multidrug-resistant gram-negative bacteria, such as colistin and carbapenem-resistant *Klebsiella pneumoniae*, represent a major problem for healthcare systems worldwide and have a high lethality rate (Capone et al., 2013; Sabino et al., 2019). In 2009, due to the increase and spread of several multidrug-resistant bacteria around the world, *K. pneumoniae* along with other multidrug-resistant pathogens (*Enterococcus faecium, Staphylococcus aureus, Acinetobacter baumannii, Pseudomonas aeruginosa, and Enterobacter spp.*) were classified as “ESKAPE,” which are the most frequent microorganisms in healthcare-associated infections in the United States that also tend to “escape” from antimicrobial treatments (Rice, 2008; Boucher et al., 2009). The Interagency Coordination Group on Antimicrobial Resistance reported in 2019 that multidrug-resistant bacteria could be responsible for the death per year of 10 million people worldwide in 2050. Currently, at least 700,000 people die a year worldwide due to multidrug-resistant bacteria (Interagency Coordination Group on Antimicrobial Resistance [IACG], 2019).

Carbapenem-resistant *Enterobacterales* generally have carbapenemase enzymes that also confer resistance to most penicillins and cephalosporins (Bratu et al., 2005). In recent years, polymyxins (polymyxin B and polymyxin E/colistin) have become an alternative treatment for carbapenem-resistant *Enterobacterales* infections and, as a result, the emergence of polymyxin resistance has increased (Falagas and Kasiakou, 2005; Gonçalves et al., 2016; Karaiskos et al., 2017).

Both polymyxin B and colistin resistance occur through two main mechanisms: (1) chromosome mediated intrinsic changes in the two components responsible for the formation of lipopolysaccharides (LPS), including the PhoPQ and PmrAB regulatory system and the *mgrB* regulation; and (2) plasmid-mediated related to *mcr* genes that modify LPS (Ah et al., 2014). Plasmid-mediated resistance to polymyxins has been identified in different regions of the world (Liu et al., 2016; Xavier et al., 2016). However, most colistin-resistant *K. pneumoniae* isolates show modifications in chromosomal genes, mainly due to alterations in the *mgrB* gene (Duin and Doi, 2015).

The lack of studies that characterize the mechanisms of resistance to carbapenems and colistin in *K. pneumoniae* (ColR-CRKP) in Latin America, in addition to the high lethality of the associated infections, make this subject of high relevance. In this study, we report a prolonged outbreak of ColR-CRKP in a large tertiary hospital in Salvador, Brazil. We describe the clinical and demographic characteristics of the patients, and microbiological and molecular investigations conducted into this outbreak.

## Materials and Methods

### Setting

The hospital is a 650-bed tertiary public referral center. The intensive care units (ICUs) comprise general, surgical, neurology, cardiology, pediatric, and neonatology units with 100 beds. From September 2016 to February 2018, patients that presented infection or colonization by ColR-CPKP identified by Vitek^®^2 at the study hospital were included in the study. Patients that the isolates did not confirm resistance to polymyxins at the laboratory of the Federal University of Bahia (UFBA) determined by the broth microdilution assay (BMD), and isolates that could not be identified, were excluded.

### Medical Records and Hospital Infection Control Committee Data

Patients infected or colonized by ColR-CRKP had their medical records reviewed using a semi-structured questionnaire that addressed sociodemographic variables (sex and age), clinical data (signs of infection, infection topography, comorbidities, invasive procedures, and use of antibiotics), laboratory data (type of ColR-CRKP culture, date of culture and infection outcome in 30 days), period of hospital stay and the sector of acquisition of ColR-CRKP. Patients’ comorbidities were assessed using the Charlson score. Patients were considered infected if they presented signs and symptoms of infection related to the culture site three calendar days prior to or after the date of the first positive diagnostic test (Horan et al., 2008).

Data from the Hospital Infection Control Committee, such as internal communications and meetings, were evaluated to describe the investigation and actions related to the ColR-CRKP cases.

### Microbiological Methods

According to the hospital protocol, all patients were submitted to rectal swab surveillance cultures on admission to the ICU, and weekly surveillance until ICU discharge. Cultures were processed according to the CDC laboratory protocol published in 2009 (Centers for Disease Control and Prevention [CDC], 2009).

All bacterial isolates identified as ColR-CRKP by the Vitek^®^2 system at the hospital laboratory were sent to the Federal University of Bahia (UFBA) to determine the minimum inhibitory concentration (MIC) for colistin by the broth microdilution assay (BMD), according to the criteria established by CLSI. Those isolates with MIC ≤ 2 μg/mL were considered sensitive to colistin and resistant if MIC > 2 μg/ml (Clinical & Laboratory Standards Institute [CLSI], 2021). The susceptibility test for aminoglycosides, piperacillin-tazobactam, cefepime, carbapenems, ciprofloxacin and ampicillin-sulbactam was performed using Vitek^®^2 and disk diffusion was used for ceftazidime-avibactam according to the criteria established by CLSI (Clinical & Laboratory Standards Institute [CLSI], 2021). Meropenem-vaborbactam was not tested because this antibiotic is not available in our country.

### Molecular Methods

#### Identification of Genes Encoding Antimicrobial Resistance

DNA extraction was carried out by boiling method as described previously by Dallenne et al. (2010). Briefly, the colonies were suspended in 100 μl of sterile ultrapure water and heated at 95°C for 10 min, followed by centrifugation at 12,000 rpm for 2 min. The supernatant was transferred to a sterile tube and stored at −20°C until PCR assays (Dallenne et al., 2010).

Carbapenem resistance genes were detected using conventional multiplex PCR for the *bla*_*KPC*_, *bla*_*VIM*_, *bla*_*OXA–*48–LIKE_, and *bla*_*GES*_ genes and two simplex PCRs for the *bla*_*NDM*_ and *bla*_*IMP*_ genes (Dallenne et al., 2010; Poirel et al., 2011; Zwaluw et al., 2015). To evaluate colistin resistance, the *mcr-1* gene and the integrity of the *mgrB* gene were investigated through simplex PCR assay. The primers and protocols used were proposed by Cannatelli et al. (2013) and Rebelo et al. (2018), respectively.

#### Pulsed-Field Electrophoresis Technique and Multilocus Sequence Typing

The evaluation of the genetic relationship between *K. pneumoniae* isolates was performed using PFGE and MLST. In the PFGE analysis, bacterial DNA was prepared and cleaved with 20 U *Xba*I endonuclease (New England Biolabs, Boston, MA, United States). Electrophoresis was performed in a 1.2% agarose gel (BMA Products, Rockland, ME, United States) prepared and ran in 0.5 × Tris-borate-EDTA buffer on a CHEF-DR III apparatus (Bio-Rad Laboratories, Richmond, CA, United States). The initial switch time was 1 s, the final switch time was 40 s, and the run time was 18 h at 6 V/cm. Gels were then stained in ethidium bromide, destained in distilled water, and photographed (GelDoc 2000; Bio-Rad). GelCompar II^®^ software (Applied Maths – bioMeriéux), with a Dice coefficient and a tolerance of 1.5, was used to determine the similarity and construct a dendrogram. MLST was performed by amplifying seven housekeeping genes (*gapA, infB, mdh, pgi, phoE, rpoB*, and *tonB*) on a selected number of ColR-CRKP isolates as previously described (Clinical & Laboratory Standards Institute [CLSI], 2021). The MLST website assigned the allele number and sequence type (ST)^1^.

#### Sequencing of the *mgrB* Gene

Sanger sequencing of the *mgrB* gene was performed using an ABI 3500 XL Genetic Analyzer (Applied Biosystems) with a BigDye Terminator v3.1 Ready Reaction Mix (Applied Biosystems) according to the manufacturer’s instructions. Sequences were edited using the Sequence Scanner Software v2.0, and the alignment with the reference *mgrB* gene of *K. pneumoniae* MGH 78578 (CP000647.1) was carried out using the Geneious Prime 2020.1.2^2^.

#### Whole-Genome Sequencing and Genomic Analysis

Whole-genome sequencing was carried out using the Illumina NextSeq 500 platform (San Diego, CA, United States). The raw sequences were trimmed using TrimGalore v.0.6.4 to remove adapters and low-quality regions (Phred quality cutoff: 20). The genome was *de novo* assembled into contigs using Unicycler (v0.4.8, 2017) (Wick et al., 2017). The resistome was predicted using the Resistance Gene Identifier (RGI) from the Comprehensive Antibiotic Resistance Database (CARD^3^) (Alcock et al., 2020). Mutations in genes potentially involved in polymyxin resistance (*pmrA*, *pmrB*, *phoP*, *phoQ, crrA, crrB*, and *mgrB*) were investigated by alignment using the BLASTn tool^4^ and Geneious Prime 2020.1.2 (see text footnote 2). To identify insertion sequences (IS), we used the online platform ISfinder^5^. The PROVEAN platform^6^ was used to predict the impact of the mutation on the biological functions of the proteins. *K. pneumoniae* MGH 78578 (GenBank accession no. CP000647.1), a polymyxin-susceptible strain, was used as a reference.

### Statistical Analysis

The Epiinfo version 3.5.1 (CDC, Atlanta, GA, United States) was used for data management and analysis. Descriptive analyses were performed, such as frequency, measures of central tendency, and percentiles. ColR-CRKP cases were stratified into episodes of infection and colonization. Absolute and relative frequencies were determined for categorical variables (a few of which are sex, previous use of polymyxins, and results of molecular tests). For non-categorical variables (e.g., Age and Charlson score) the median and interquartile ranges were calculated. The Mantel-Haenszel chi-square statistical probability test or Fisher’s exact test were used to assess statistical significance. Mantel-Haenszel test was used to compare continuous variables. For univariate analyses, statistical significance was defined as *P* < 0.05

### Ethical Aspects

The study was approved by the Hospital Research Ethics Committee (CEP) on April 22, 2018, authorization number: 79256117.2.0000.5028.

## Results

**FIGURE 1:**
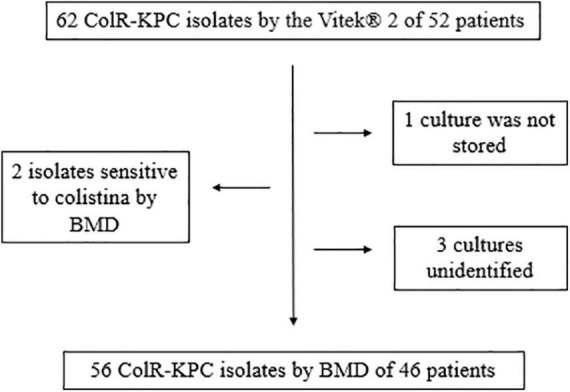
Flowchart of ColR-CRKP positive culture cases identified during the study period.

The first patient with a positive culture of ColR-CRKP was identified on 25 September 2016, followed by 46 additional patients occurring between September 2016 and February 2018. The total number of patients screened with rectal swabs during this interval was not available. A total of 56 ColR-CRKP cultures from 46 patients were analyzed according to the flowchart described in Figure 1. Due to logistical issues, we obtained clinical and demographic data only from 31 of the 46 patients (67%) included in the study.

### Index Case

On 25 September 2016, the first ColR-CRKP isolate was identified in a catheter tip culture from a patient from the general ICU. The index patient was 45 years old and had been hospitalized for an alteration of consciousness. She underwent orotracheal intubation, mechanical ventilation, and a central venous catheter. She received meropenem and polymyxin for 16 days and vancomycin for 17 days before the identification of ColR-CRKP. The duration of ICU hospitalization was 24 days until the ColR-CRKP isolate identification. The isolate from the index case was not stored for further analysis.

### Description of the Study Patients

Table 1 depicts the patients’ characteristics and outcomes. In general, patients were male (67.7%), with a median age of 62 years, the youngest being 20 and the oldest 86 years. The median of the Charlson score was 3. The main comorbidities were systemic arterial hypertension (38.7%), diabetes (32.2%), and cerebrovascular disease (25.8%). No patient had any pharmacological immune suppression. The average hospitalization stay until ColR-CRKP identification in days were 35,12, the minimum 5 and the maximum 131 days.

**TABLE 1:** Clinical characteristics of ColR-CRKP patients identified during prolonged outbreak at a large tertiary hospital in Brazil (*n* = 31).

Characteristics	*n* (%)
**Sex**	
Male	21 (67.7)
Female	10 (32.3)
**Age (years)**	
Median age (IQ)	62
Minimum	20
Maximum	86
**Charlson score**	
Median	3
**Average hospitalization stay until ColR-CRKP identification (days)**	35,12
	(5 – 131)
**Comorbid conditions**	
Systemic arterial hypertension	12 (38.7)
Diabetes	10 (32.2)
Cerebrovascular disease	8 (25.8)
Congestive heart failure	2 (6.4)
Solid malignant tumor	2 (6.4)
Chronic liver disease	2 (6.4)
Chronic lung disease	1 (3.2)
Acute myocardial infection	1 (3.2)
Hematological malignancy	1 (3.2)

The majority of the patients underwent mechanical ventilation (90.3%) and central venous catheter (93.5%), and 18 (58%) had any surgery or bladder catheterization during hospitalization. A total of 38.7% (*n* = 12/31) were infected by ColR-CRKP, and 61.3% (*n* = 19/31) were colonized. Infections recorded in descending order of frequency were: surgical site infection, pneumonia, primary bloodstream infection, and urinary tract infection (Table 2). Three (25%) of the 12 infected patients had previously identified colonization.

**TABLE 2:** Risk factors and clinical outcome of patients infected and/or colonized by ColR-CRKP at a large tertiary hospital in Brazil (*n* = 31).

Interventions, infection and outcome	*n* (%)
**Procedures**	
Central venous catheter	29 (93.5)
Mechanical ventilation	28 (90.3)
Orotracheal intubation	26 (83.8)
Bladder catheter	18 (58.0)
Surgery	18 (58.0)
Invasive mean arterial pressure	17 (54.8)
Nasogastric tube	26 (46.4)
Peripheral venous catheter	13 (41.9)
Ventricular shunt	6 (19.3)
Colonized patients	19 (61.3)
Infected patients	12 (38.7)
Surgical site infection	5 (41.6)
Pneumonia	4 (33.4)
Bloodstream infection	2 (16.7)
Urinary tract infection	1 (8.3)
**Outcomes in infected patients (*n* = 12)**	
Deaths due to ColR-CRKP	7 (58.4)
Cure	3 (25)
Other cause of deaths	1 (8.3)
Transfered/others	1 (8.3)

Regarding the use of antibiotics before the isolation of ColR-CRKP, 70.9% used carbapenem with an average use of 13.2 days preceding the first positive culture. A total of 32.2% (*n* = 10/31) used polymyxin, with an average of 15.1 days of use, and 35.5% (*n* = 11/31) used carbapenem and polymyxin before the isolation of ColR-CRKP.

The lethality of ColR-CRKP infection was 58.4% (*n* = 7/12). The infections that were associated with the patients’ deaths were surgical site (5 cases), bloodstream infections associated with a central venous catheter (2 cases), pneumonia (4 cases), and urinary tract infection (1 case). No other co-infections were detected in those patients. One patient died from another cause and another was transferred. Among the seven patients that have died because of ColR-CRKP, the median Charlson score was 3, IQ (2–5), and in the three survivors the median was also 3, IQ (1–4).

### Description of Isolates

The 56 ColR-CRKP isolates analyzed were from 46 patients, 29 patients from the general ICU and 17 patients from different sectors of the hospital. All isolates were identified in patients hospitalized for more than 48 h.

Among the 56 ColR-CRKP isolates, 67.8% (38 isolates out of 56) of the cultures were from surveillance rectal swabs, which were collected in the weekly routine of the intensive care units or at patient admission. Among clinical cultures, the most prevalent was blood culture in 8.9% (*n* = 5), followed by catheter tip (7.1%, *n* = 4), intraoperative secretion (7.1%, *n* = 4), tracheal secretion (5.3%, *n* = 3), and urine culture (3.6%, *n* = 2).

More than half (55%) had a MIC of 256 μg/mL to colistin, and the MIC90 was > 16 μg/mL. Regarding the antimicrobial resistance of ColR-CRKP isolates to other antibiotics, we identified resistance to amikacin (3.6%), gentamicin (91.1%), piperacillin-tazobactam (95.8%), cefepime (98.1%), ciprofloxacin (98.1%), and ampicillin-sulbactam (100%). We did not find ceftazidime-avibactam resistance in KPC-producing isolates.

The carbapenemase gene *bla*_*KPC*_ was the most common and was found in 94.7% (*n* = 53/56) of the isolates, followed by the *bla*_*NDM*_ gene in 16% (*n* = 9/56) and the *bla*_*GES*_ gene in 1.7% (*n* = 1/56). Co-harboring carbapenemase genes were found, with *bla*_*KPC*_ and *bla*_*NDM*_ detected in six isolates (10.7%) and *bla*_*KPC*_ and *bla*_*GES*_ in one isolate (1.7%). The presence of other metallo-ß-lactamases genes, such as *bla*_*VIM*_, *bla*_*IMP*_, and *bla*_*OXA–*48_, were not detected.

The presence of *mcr-1* was not identified in all isolates. Regarding the mechanisms of colistin resistance, PCR for the *mgrB* gene was performed in all isolates. An alteration in size of the *mgrB* gene was detected in 69.7% (*n* = 39/56). The *mgrB* gene was unchanged in size in 21.4% (*n* = 12/56) and in 8.9% (*n* = 5/56), *mgrB* gene was not detected. All isolates that had alterations in the size of the *mgrB* gene had a similar size in the PCR gel (≥900 bp).

**FIGURE 2:**
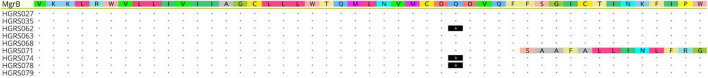
Alignment of the amino acid sequences of *mgrB* from ColR-CRKP strains that were not disrupted by insertion sequences. Dots indicate identical residues; asterisk indicates stop codon; letter indicates amino acid substitution. The first sequence in the alignment belongs to the wild-type strain *Klebsiella pneumoniae* MGH 78578 (CP000647.1).

The *mgrB* gene sequencing was performed in nine isolates with altered size of the *mgrB*, chosen at random, and all of them were disrupted by insertion sequence IS*903B* (95% nucleotide identify; IS5 family). Nine unchanged *mgrB* genes were also sequenced to detect point mutations. The premature stop codon (Q30*) was detected in three isolates. We also identified in one isolate a single nucleotide deletion (thymine deletion at position 104 – 104delT), causing a frameshift downstream of amino acid 35 (F35fs) (Figure 2). In summary, alterations in the *mgrB* gene were detected in 87.5% (49/56) of isolates, while no changes were identified in the remaining isolates (12.5%, 7/56).

Whole-genome sequencing resistome analysis was performed in one representative ColR-CRKP isolate from the patient 28 identifying sequence type (ST) 11. We have detected the following deleterious mutations associated with colistin resistance: R256G in PmrB and disruption of the *mgrB* gene by IS*903B*. We also found T246A and C68S mutations in PmrB and CrrB, respectively; however, they were predicted to be neutral by the PROVEAN platform. Alterations in the *pmrA*, *phoP*, *phoQ*, and *crrA* genes were not detected (Table 3). The *bla*_*KPC–*2_ gene, located on a Tn*4401* isoform “b” transposon, was detected in this sequenced isolate. Other antimicrobial resistance genes are listed in Table 4.

**TABLE 3:** Mutation analysis of genes involved in colistin resistance of one ColR-CRKP sequenced isolate.

Isolate	PmrA	PmrB	PhoP	PhoQ	CrrA	CrrB	*mgrB*	*mcr-1*
HGRS058	WT	T246A^a^ R256G^b^	WT	WT	WT	C68S^a^	Gene disrupted by IS*903B*^b^	ND

*ND, not detected; WT, wild type. ^a^Mutation predicted as neutral by PROVEAN. ^b^Mutation predicted as deleterious by PROVEAN.*

**TABLE 4:** Predicted resistome other than colistin resistance genes of one ColR-CRKP sequenced isolate (HGRS058).

Resistance mechanism	AMR gene family	AMR gene	SAPs
Antibiotic inactivation	β-lactamases	*bla*_*TEM–*1_, *bla*_*SHV–*11_, *bla*_*KPC–*2_, and *ampH*	
	Fosfomycin thiol transferase	*fosA6*	
	AAC(3)	*aac(3)-IIe*	
Antibiotic efflux	ATP-binding cassette (ABC) antibiotic efflux pump	*msbA* and *lptD*	
	Major facilitator superfamily (MFS) antibiotic efflux pump	*emrR*, *kpnE*, *kpnF*, *kpnG*, and *kpnH*	
	Major facilitator superfamily (MFS) antibiotic efflux pump, resistance-nodulation-cell division antibiotic efflux pump	H-NS	
	Resistance-nodulation-cell division (RND) antibiotic efflux pumps	*oqxA* and *oqxB*, *marA*, *adeF*, *CRP*, *baeR*, and *rsmA*	
Antibiotic target alteration	Pmr phosphoethanolamine transferase	*eptB* and *arnT*	
	Antibiotic-resistant UhpT	*uhpT* with mutation	E350Q
	Elfamycin resistant EF-Tu	EF-Tu with mutation	R234F
	Penicillin-binding protein mutations conferring resistance to beta-lactam antibiotics	PBP3 with mutation	D350N, S357N
	Fluoroquinolone resistant *gyrA*	*gyrA* with mutation	S83I
	Fluoroquinolone resistant *parC*	*parC* with mutation	S80I
Antibiotic target replacement	Sulfonamide resistant sul	*sul1*	
	Trimethoprim resistant dihydrofolate reductase	*dfrA30*	
Antibiotic efflux, antibiotic target alteration	Resistance-nodulation-cell division (RND) antibiotic efflux pumps	*marR* with mutation	
Reduced permeability to antibiotic	Bacterial porins	*ompK37* and *ompA*	

*AMR, antimicrobial resistance; SAP, single amino acid polymorphism.*

**FIGURE 3:**
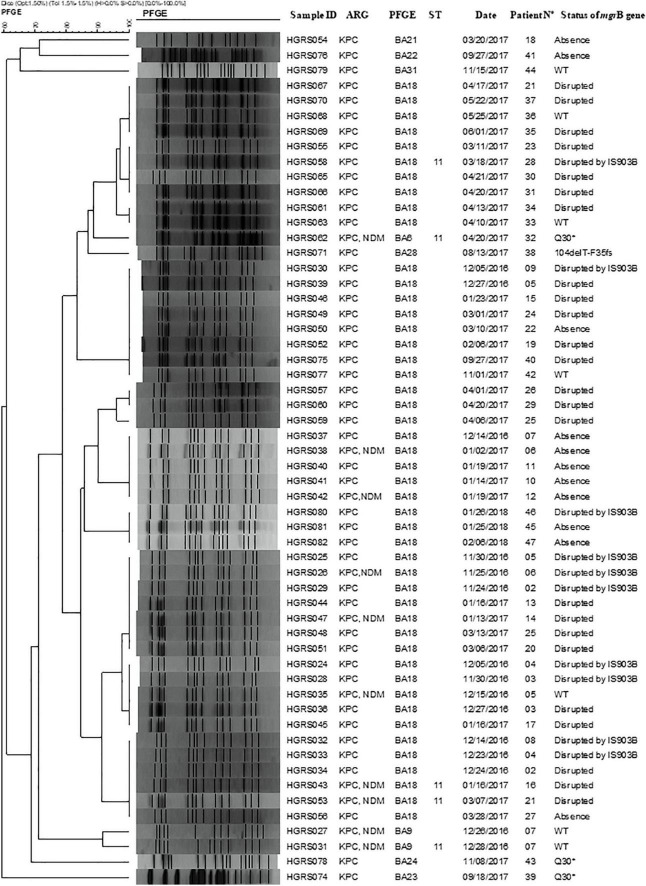
Dendrogram showing the relatedness of ColR-CRKP based on PFGE patterns after digestion with *Xba*I. Isolates showing 80% similarity are considered related. ARG, Antimicrobial Resistance Genes; ID, identification; KPC, Klebsiella pneumoniae carbapenemase; NDM, New Delhi metallo-beta-lactamase; PFGE, Pulsed-field electrophoresis technique; ST, sequence typing; WT, Wild type; fs, Frameshift.

**FIGURE 4:**
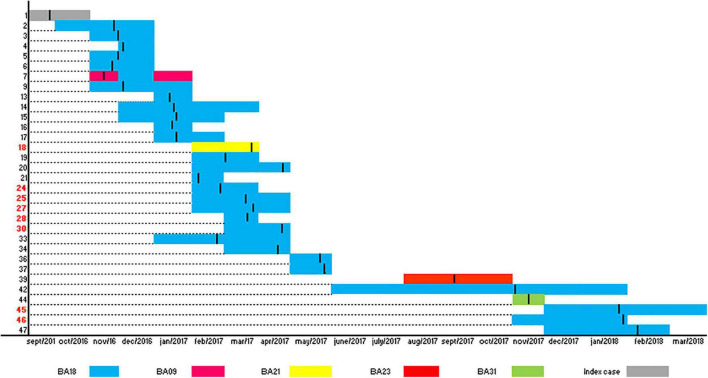
Timeline and transmission opportunities among patients during the outbreak including the index patient according to the PFGE profile, culture date and period of hospitalization. The 31 patients who had their medical records evaluated and the index case were placed on the *Y* axis. The numbers in black on the *Y*-axis indicate patients who were admitted to the general ICU and in red patients from other sectors of the hospital (24: UAVC; 25: emergency; 27: medical clinic ward; 28: emergency ICU; 30: emergency ICU; 45: emergency ICU and 46: ICU: neurological). The length of stay was placed monthly on the *X*-axis. Each bar indicates a patient, and the length of the bar signals the length of stay. The transverse lines on each bar indicate the moment of identification of ColR-CRKP. The color of the bars indicates the PFGE profile.

PFGE analysis of ColR-CRKP isolates identified nine PFGE patterns (Figure 3). The PFGE BA18 patterns was found in 47 isolates. The PFGE BA9 patterns was detected in two isolates, which belonged to the same patient, and another seven PFGE patterns (BA23, BA31, BA6, BA22, BA28, BA21, and BA24) were detected in only one isolate each. MLST typing of major PFGE patterns belonged to sequence type (ST11). The clonal relationship of PFGE patterns is shown in Figure 3 and the timeline and transmission opportunities among patients during the ColR-CRKP outbreak, including the index patient in Figure 4.

The PFGE BA18 pattern was initially detected in the general ICU of the hospital and later spread out to different sectors of the hospital, including the emergency sector. Of the patients who acquired ColR-CRKP in other sectors, whose medical records could be evaluated, 85.71% (*n* = 6/7) underwent a surgical procedure before identifying the ColR-CRKP culture, and the same proportion did not use polymyxin prior to the first culture. None of these patients had a report of previous hospitalization in other hospitals in the last 6 months.

No statistical association was found regarding sex, age, type of carbapenemase, change in *mgrB* gene, previous use of polymyxin, Charlson score, or previous surgery between infected and colonized patients (Table 5). Three of the 12 infected patients that had previously identified colonization had all isolates belonged to PFGE BA18.

**TABLE 5:** Comparative analysis between patients infected and who colonized by ColR-CRKP (*n* = 31).

Characteristics	Infection (*n* = 12) (%)	Colonization (*n* = 19) (%)	*p-value*
Sex (*n* = 31)			
Male	7 (58,3)	14 (73,7)	0.31*
Age^1^ (*n* = 31)	56,5 (30–75)	62 (51–70)	0.61+
*bla*_*KPC*_	11 (91,7)	18 (94,7)	0.63*
*bla*_*NDM*_	1 (8,3)	3 (15,8)	0.49*
Alteration in *mgrB* gene	10 (83.3)	16 (84.2)	1.0*
Prior use of Polymyxin	5 (41,7)	5 (26,3)	0.31*
Previous surgery	9 (75,0)	10 (52,6)	0.19*
Charlson Score	3 (1–4)	4 (2–6)	0.21+

^1^Median, interquartile range. * Exact fisher test. + Mann-Whitey/Wilcoxon test.

### Description of Hospital Infection Control Interventions

**FIGURE 5:**
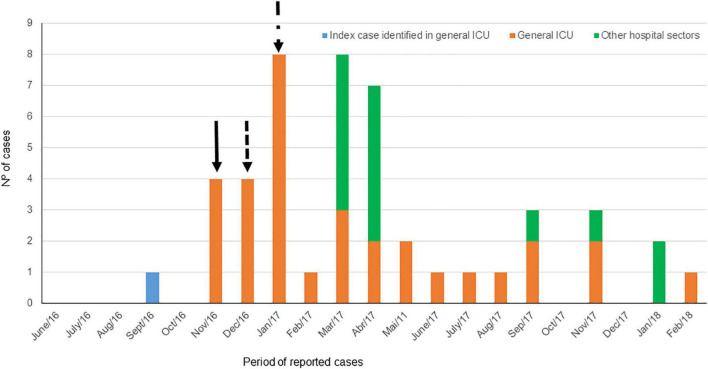
Temporal distribution of 46 patients with ColR-CRKP and the index case according to the PFGE profile and intervention points performed in the hospital. Full black line: removal of bed curtains, intensification of cleaning of the environment, greater availability of alcohol gel for hand hygiene, removal of adornments from health professionals, introduction of rectal swab at the admission of patients to the sector and daily control of prescription of antimicrobials. Black dotted line: functional cohort of staff for patients colonized/infected by ColR- CRKP, intensification of hand hygiene, universal contact precaution in all patients admitted to the ICU and weekly rectal swab in all patients (in addition to the swab rectal and nasal surveillance at admission of all patients). Dashed black line with dot: physical restructuring of the unit, removal of damaged mattresses, terminal cleaning twice (by different hygiene teams) of the beds of patients who were discharged or died before the hospitalization of another patient.

The Hospital Infection Control Committee carried out several interventions to mitigate the ColR-CRKP outbreak, as described in Figure 5. Concerning the care environment, the general ICU did not have an extra curtain to replace those that separated each bed in the ICU, remaining for weeks without being removed. Considering that the curtains do not allow adequate cleaning, it was decided to replace them with fixed screens that enabled better hygiene. Another critical intervention was the removal of all damaged mattresses of the hospital, which made it difficult to properly clean. The Hospital Infection Control Committee isolated all ColR-CRKP cases from the general ICU in a semi-intensive unit after ICU discharge to prevent the dissemination of the strain throughout the hospital.

## Discussion

This study presents 56 cases of ColR-CRKP isolates detected in a large tertiary hospital from September 2016 to February 2018. Most of them were from the general ICU and have probably been disseminated to different hospital sectors. Molecular analysis showed that 83.9% (*n* = 47/56) of the isolates belonged to the same PFGE profile, confirming the occurrence of an outbreak. Since polymyxin was not used by 67.6% (*n* = 21/31) of the patients and the isolates were predominantly from the same cluster, cross-transmission was probably the leading cause of ColR-CRKP spread. This result conflicts with a previous study carried out in São Paulo, Brazil, which suggested that the ColR-CRKP cases might have been a consequence of selective pressure due to the increased use of polymyxins and not because of horizontal transmission, since several clusters were identified in PFGE and the search for the *mcr-1*/*mcr-2* genes were negative (Boszczowski et al., 2019).

Previous Hospital Infection Control Committee data reports show that between 2015 and 2016, more than 70% of the *Enterobacterales* in the general ICU were not susceptible to carbapenems. As a result, combination therapy with polymyxin and carbapenem was recommended as the empirical treatment for sepsis, which might have contributed to the emergence of the ColR-CRKP strain. Other factors, such as environmental non-conformities and healthcare professionals who worked in different sectors simultaneously or consecutively, have probably facilitated the spread of ColR-CRKP. High consumption of carbapenems and colistin, inadequate hospital structure, and low adherence to infection control measures have already been related to the emergence and spread of the ColR-CRKP strains (Gonçalves et al., 2016).

Alteration of the *mgrB* gene was detected in 87.5% (*n* = 49/56) of the isolates, and of these, 49.0% (24/49) had an alteration in size probably due to IS*903B*, 22.4% (11/49) did not have the *mgrB* gene detected, 20.4% (10/49) presented the IS*903B*, 6.1% (3/49) had a premature stop codon (Q30*), and 2.1% (1/49) presented a thymine deletion at position 104 – 104delT (F35fs). The inactivation of the *mgrB* gene can occur through numerous mutations, such as deletion and insertion. The *mgrB* gene is responsible for the negative regulator of the PhoPQ, and its inactivation results in the addition of cationic groups on LPS, reducing the affinity to polymyxins (Poirel et al., 2015; Rodrigues et al., 2019). Except for the sequenced strain, our study has only investigated the chromosomal *mgrB* gene and the presence of the *mcr-1* gene, genetic alterations in the *mgrB* gene seem to be the main factor responsible for resistance to polymyxins in Brazil and worldwide (Poirel et al., 2015; Braun et al., 2018; Pitt et al., 2018). No *mcr-1* was detected in our isolates. The detection of *mcr* genes in *K. pneumoniae* appears to be sporadic in Brazil, as few studies have reported the isolation of *mcr-1* positive ColR-CRKP strains to date (Aires et al., 2017; Dalmolin et al., 2018; Higashino et al., 2019).

The deleterious mutation Q30* in *mgrB* was already reported in polymyxin-resistant *K. pneumoniae* isolated from Brazil (Aires et al., 2016). There is *in vivo* evidence that this mutation is responsible for colistin resistance in clinical strains of *K. pneumoniae* (Kong et al., 2021). In the literature, only one study reported the frameshift mutation 104delT - F35fs (Aulin et al., 2021).

The MIC90, the concentration that inhibited 90% of the strains in our study, was > 16 μg/ml, and more than half of the isolates (55%) had a MIC of 256 μg/ml. In an Iranian study, in which the *mgrB* gene mutation was primarily responsible for polymyxins resistance, all strains that identified changes in the *mgrB* gene sequence also had high MICs, all of which were > 64 μg/ml (Haeili et al., 2017). Other study have also described elevated MIC in isolates with altered *mgrB* gene (Pitt et al., 2018).

The sequencing of the ColR-CRKP strain from patient 28 evidenced the *bla*_*KPC–*2_ gene, ST-11, and the MLST analysis, identified four other isolates as ST-11. ST-11 is the most commonly found ST in some regions of the world, such as China, and appears to have advantages in terms of survival time on intensive care unit surfaces compared to other STs of *K. pneumoniae*. This possible feature may contribute to its wide dissemination (Liu et al., 2020).

The WGS resistome analysis has also found two deleterious mutations, R256G in PmrB and disruption of the *mgrB* by IS*903B*, the same IS detected in the other *mgrB* genes sequenced. The *mgrB* gene disruption, mainly mediated by IS5-like elements, is a common chromosomal mechanism of polymyxin resistance in *K. pneumoniae* (Cannatelli et al., 2013; Poirel et al., 2015). While predicted as a deleterious mutation by PROVEAN, the R256G in PmrB is not related to polymyxin resistance (Cheng et al., 2015).

T246A and C68S mutations in PmrB and CrrB were also detected. T246A in PmrB, predicted as a neutral mutation, was found in polymyxin-susceptible isolates (Aulin et al., 2021). The C68S mutation in CrrB, also predicted to be neutral, was already reported in previous studies (Longo et al., 2019; Baron et al., 2021; De La Cadena et al., 2021). The C68S mutation is frequently associated with additional mutations in other polymyxin resistance genes. However, a study in Brazil reported two mutations in one isolate with colistin resistance (MIC = 32 μg/mL), R256G in PmrB (not related to polymyxin resistance), and C68S in CrrB (Longo et al., 2019). It is noteworthy that the authors did not investigate mutations in the *crrA* gene. Although apparently responsible for polymyxin resistance, studies should investigate the impact of the C68S mutation on CrrB function.

The lethality of 58.4% of the ColR-CRKP-infected patients in our study was similar to that of another study from São Paulo, whose overall lethality within 30 days was 64 and 53%, respectively (Boszczowski et al., 2019). Another Brazilian study showed that the lethality of patients infected with ColR-KPC strains was 44.4% (Gonçalves et al., 2016). In an Italian study, ColR-CRKP lethality was 40.6%, and multivariate analysis demonstrated that colistin resistance was an independent risk factor for mortality (Capone et al., 2013).

One of the limitations of the study was the use of Vitek^®^2 in screening, which is not recommended to assess sensitivity to polymyxins due to its high false sensitivity. The misplaced samples and non-collection of cultures might have reduced the number of strains in the study. Another relevant limitation was the low number of patients and that the hospital did not have an electronic medical record, which made data collection difficult, as many medical records were not found or were incomplete, preventing other patients from being categorized as infected. Regarding resistance to polymyxins, except for the sequenced strain, our study investigated only the *mgrB* and the *mcr-1* genes.

## Conclusion

This study presents a prolonged outbreak of ColR-CRKP in which 83.9% of the isolates belonged to the same cluster and 67.6% of the patients evaluated had not used polymyxin, suggesting the possibility of cross-transmission of ColR-CRKP isolates with inactivation of the *mgrB* gene identified in 87.5%.

## Data Availability Statement

The datasets presented in this study can be found in online repositories. The names of the repository/repositories and accession number(s) can be found at: GenBank, JAJPGK000000000.

## Ethics Statement

The studies involving human participants were reviewed and approved by the Institutional Review Boards of Hospital Geral Roberto Santos (CAAE n° 79256117.2.0000.5028). Written informed consent for participation was not required for this study in accordance with the national legislation and the institutional requirements.

## Author Contributions

VR, HL, NS, AMa, LA, and JR contributed to the conceptualization of the research project and collected samples, and clinical information. MB and GS carried out molecular tests. VR performed the clinical exams. VR and JR wrote first draft of the manuscript. VR, JA, and AMo analyzed and interpreted the data. HL, BM, MR, and FB critically reviewed and edited the manuscript. JR was responsible for the project administration and supervised all procedures executed in this research. All authors have read and approved the final manuscript.

## Conflict of Interest

The authors declare that the research was conducted in the absence of any commercial or financial relationships that could be construed as a potential conflict of interest.

## Publisher’s Note

All claims expressed in this article are solely those of the authors and do not necessarily represent those of their affiliated organizations, or those of the publisher, the editors and the reviewers. Any product that may be evaluated in this article, or claim that may be made by its manufacturer, is not guaranteed or endorsed by the publisher.

## Footnotes

1
http://bigsdb.pasteur.fr/klebsiella/

2
https://www.geneious.com

3
https://card.mcmaster.ca/analyze/rgi

4
https://blast.ncbi.nlm.nih.gov/Blast.cgi

5
https://isfinder.biotoul.fr/

6
http://provean.jcvi.org/index.php
